# Enhanced antimicrobial efficacy of a vancomycin/zinc oxide/chitosan nanocomposite via *Bacillus licheniformis*-mediated biomodification

**DOI:** 10.1186/s11671-025-04398-1

**Published:** 2025-12-07

**Authors:** Mohamed I. Abou-Dobara, Zakaria A. M. Baka, Shimaa M. El-Salamony, Mohamed M. El-Zahed

**Affiliations:** Department of Botany and Microbiology, Faculty of Science, Damietta University, New Damietta, 34517 Egypt

**Keywords:** Antimicrobial activity, *Bacillus licheniformis*, Nanocomposite, Vancomycin, ZnO NPs

## Abstract

Recognition of antimicrobial resistance (AMR) is crucial for a strong publication. Drug-resistant microbes, such as *Candida albicans*, methicillin-resistant *Staphylococcus aureus* (MRSA), *Escherichia coli*, *Proteus mirabilis*, and *Klebsiella pneumoniae*, pose a significant health threat. There is an urgent need for innovative and synergistic therapies. The new engineered nanocomposite system, zinc oxide/chitosan nanocomposite loaded with vancomycin (VA/ZnO/CS), directly addresses this challenge by aiming to enhance or restore the efficacy of existing drugs. Zinc oxide nanoparticles (ZnO NPs) were biosynthesized using *Bacillus licheniformis* ATCC 4527, and then combined with chitosan (CS) and vancomycin (VA) through a green chemical method. The nanocomposite that was produced was characterized using various techniques. The results of UV–Vis spectroscopy showed an adsorption peak at 348 nm. The material matrix of the nanocomposite contains ZnO NPs and numerous active groups, as indicated by the results of X-ray diffractometer (XRD) and Fourier transform infrared spectroscopy (FTIR). Images captured by transmission electron microscopy (TEM) showed that the VA/ZnO/CS particles were spherical with an average size of 78 ± 2.3 nm. The mean crystallite size of the nanocomposite was calculated using the Scherrer equation from the XRD data (79.38 nm) which closely matched the dimensions of the ZnO core observed in the TEM images (78 ± 2.3 nm). The antimicrobial activity of VA/ZnO/CS was tested against *Bacillus cereus* ATCC 14,579, MRSA ATCC 33,592, *P. mirabilis* AUF1, *Klebsiella pneumoniae* ATCC 11,296, and *Candida albicans* ATCC 10,231. Compared to common drugs like fluconazole and vancomycin, VA/ZnO/CS demonstrated significantly higher levels of biocidal activity in the agar well-diffusion test, minimum inhibitory concentration (MIC), and minimum microbicidal concentration (MMC). The antimicrobial activity was found to be dependent on the dose of nanocomposite with higher doses resulting in increased antimicrobial inhibition. The prepared nanocomposite achieved a complete biocidal effect against the investigated microorganisms with 5–15 µg/ml, while conventional drugs required 25–30 µg/ml. The powerful antimicrobial action of VA/ZnO/CS was demonstrated by the TEM micrographs of *C. albicans* showing malformations and distortions of cell structure, including cell wall destruction and the emergence of vacuoles. Based on the results, the green synergy between ZnO/CS nanocomposite and VA will provide an effective biomaterial for treating infections and microbial diseases.

## Background

Antimicrobial Resistance (AMR) is universally recognized as one of the most significant and pervasive threats to human health, society, and the environment [1]. AMR occurs when pathogens develop the capacity to become resistant to drugs designed to eradicate them, consequently making infections more challenging to cure and increasing the risks of disease spread and mortality. The phenomenon is complex, driven by the interaction of genetic factors and selective pressures, largely resulting from the misuse and abuse of antibiotics in healthcare, veterinary medicine, and agriculture [2]. This process creates stronger microbial strains that spread unchecked. The clinical burden of drug-resistant diseases is already staggering, having been linked to an estimated 4.95 million deaths worldwide in 2019, with the majority affecting low- and middle-income countries (LMICs) [3]. Absent decisive action, AMR is projected to cause 10 million deaths annually by 2050 [4].

Within this crisis, fungal pathogens, particularly *Candida albicans*, represent a critical challenge [5]. While *C. albicans* is a common, asymptomatic member of the human microbiota, changes in host factors (e.g., immunosuppressive medication) or the local environment (e.g., antibiotic presence) can trigger its transition into a virulent pathogen [6]. Infections range from superficial mucosal and dermal candidiasis to life-threatening, hematogenously disseminated infections with high mortality rates (up to 40%). The clinical situation is compounded by documented microbial resistance of *C. albicans* to current anticandidal agents such as fluconazole and voriconazole, underscoring the urgent need for new therapeutic solutions [7].

In addition, pathogenic bacteria, including critical Gram-positive species like *Staphylococcus aureus*, and *Streptococcus pneumoniae* as well as Gram-negative species such as *Escherichia coli*, *Pseudomonas aeruginosa*, *P. mirabilis*, and *Klebsiella pneumoniae*, are the central drivers of the antibacterial resistance crisis [8]. These organisms employ diverse genetic and biochemical mechanisms—including the production of inactivating enzymes, modification of drug targets, and enhanced efflux pump activity—to survive antibiotic onslaught [9]. This rapid evolution has rendered many first-line antibiotics ineffective, leading to difficult-to-treat infections that prolong hospitalization and increase mortality. Furthermore, the spread of resistance is not confined to clinical settings; it is a complex, interacting phenomenon among humans, animals, and the environment. This necessitates the urgent development of novel antibacterial agents, particularly those with mechanisms of action fundamentally different from traditional small-molecule antibiotics. This is in order to circumvent existing resistance pathways and stay ahead of microbial evolution.

The limitations in tackling AMR can be broadly categorized into three areas: Scientific and Clinical, Economic and Regulatory, and Societal and Surveillance. The existing drawbacks and limitations in tackling AMR are complex and multi-layered, demanding coordinated scientific, economic, and societal interventions. Scientifically and clinically, the greatest challenges are the severely limited pipeline for genuinely novel antibiotic classes, particularly against Gram-negative pathogens, and the slow pace of current diagnostic methods, which delays targeted therapy and encourages empirical broad-spectrum use [10]. Economically and regulatorily, the main limitation stems from the unsustainable market for new antibiotics; their short course of use and the need to preserve them as last-resort drugs result in a poor return on investment for pharmaceutical developers, creating a critical funding gap that deters innovation. This issue is magnified by the insufficient use of “pull” incentives (market entry rewards) from governments [11]. Finally, on a societal and public health level, AMR is driven by the rampant inappropriate use of antibiotics in both human medicine and agriculture, coupled with fundamental failures in global hygiene, sanitation, and infection control. These practices continuously apply selective pressure while facilitating the rapid transmission of resistant strains, with surveillance efforts in many countries remaining inadequate to accurately map the full global extent of the threat [12].

To address the limitations of conventional therapeutics, researchers are increasingly focused on nanotechnology [13–17]. Nanoparticles (NPs), defined as particles ranging from 1 to 100 nanometers, possess unique size-dependent chemical properties that make them promising candidates for new treatments. NPs exhibit antibacterial, antifungal, disinfecting, and antiviral potential, suitable for diverse biological applications [18–20]. Nanomaterial synthesis employs both top-down and bottom-up methods [21]. However, conventional nonbiological methods (such as sol-gel or pyrolysis) often involve high costs, significant energy consumption, and toxic reactants [22]. The biological methods, known as “green synthesis,” provide an environmentally friendly alternative [23]. This approach utilizes microorganisms or plant extracts to biosynthesize NPs, leveraging naturally occurring biomolecules to reduce and cap metal ions, making the process biocompatible and efficient [24–27]. Probiotics, such as members of the *Lactobacillus* and *Bifidobacterium* species, are particularly valuable for biosynthesizing NPs (e.g., Ag, Cu, Zn) due to the enhanced stability and health benefits they provide [28–30]. *Bacillus licheniformis*, a recognized probiotic strain known for producing a broad range of antibiotic and immunomodulatory compounds, is a potent candidate for green synthesis [31, 32]. Its exopolysaccharides have been successfully utilized for the novel and efficient synthesis of zinc oxide nanoparticles (ZnO NPs) [33].

ZnO NPs are metal oxide NPs that are FDA-recognized as generally safe (GRAS) and are valued for their antimicrobial and regenerative properties in biomedical applications [34]. Despite their potency against pathogens like *E. coli*, *P. mirabilis*, *S. aureus*, *K. pneumoniae*, *P. aeruginosa*, and *C. albicans*, some microbes are capable of developing resistance even to NPs [35–37]. Conventional monotherapy is increasingly failing due to established and emerging resistance mechanisms. To maximize efficacy and overcome nascent resistance, the current study sought to develop a novel nanocomposite by combining ZnO NPs with vancomycin (VA) in the presence of chitosan (CS). This unique formulation was designed to harness the established antimicrobial power of ZnO and the inherent antimicrobial properties of CS. The addition of VA—a potent antibiotic—was explored for potential synergistic antimicrobial effects. This research examines the antimicrobial properties of the green synthesized vancomycin/zinc oxide nanoparticles/chitosan nanocomposite (VA/ZnO/CS) in vitro and compares its performance directly to the standard antibacterial and antifungal agents, vancomycin and fluconazole, respectively. This platform is strategically designed to bypass single-mechanism resistance by combining the reactive oxygen species (ROS) generation of ZnO NPs, the membrane disruption afforded by cationic CS, and the specific antimicrobial action of VA. The resulting nanocomposite aims to revitalize and enhance the potency of conventional medicines against a broad spectrum of pathogens, thereby offering a novel and highly relevant intervention in the fight against the AMR crisis. The overall goal is to validate this green-synthesized, multi-component material as a highly effective new candidate for broad-spectrum antimicrobial applications.

## Materials and methods

### Materials

CS (MW 50–190 KDa) ans Zn (NO_3_)_2_ (≥ 99.0%) were purchased from Sigma-Aldrich, USA. VA was purchased from Mylan Pharmaceuticals Ltd., Ireland. Fluconazole was purchased from Pfizer Inc., USA. *Bacillus cereus* ATCC 14,579, MRSA ATCC 33,592, *P. mirabilis* AUF1, *K. pneumoniae* ATCC 11,296, and *Candida albicans* ATCC 10,231 were obtained from the Microbiology Lab, at the Faculty of Science, Damietta University. Different culture media including nutrient agar, yeast extract peptone agar (YEPA), nutrient broth, yeast extract peptone broth medium (YEPB) were purchased from Oxoid, UK.

### Biosynthesis of ZnO NPs

*Bacillus licheniformis* ATCC 4527 was used to inoculate sterile nutrient broth flasks to achieve a concentration of 0.5 McFarland (1–2 × 10^8^ CFU/ml). The flasks were then incubated for 24 h. at 150 rpm and 37 °C. After the incubation period, the bacterial cultures were centrifuged at 5000 rpm for 15 min to collect cell pellets. The pellets were washed at least three times with distilled water [38]. After being reconstituted in Zn (NO_3_)_2_ solution (3 mM), the cell pellets were incubated for 24 h. at 37 °C and 150 rpm in a dark environment. The reaction mixture was then centrifuged at 4000 rpm for 15 min after incubation in order to remove the supernatant. ZnO NPs were extracted from bacterial pellets using ultrasonography after they had been cleaned at least three times with distilled water. After centrifuging the tubes for five min at 4000 rpm, the ZnO NPs-containing supernatant was poured into fresh tubes. Lastly, the ZnO NPs solution was calcined for three hrs. at 500 °C after being dried for twenty-four hrs. at 80 °C [39].

### Coating of zinc oxide with chitosan

Ten milliliters of 1% acetic acid were used to dissolve six milligrams of CS at room temperature. The mixture was then agitated for 15 min to ensure full dissolution. An Elmasonic S100H ultrasonic bath (50/60 Hz, Germany) was used to ultrasonically treat 6 mg of ZnO NPs at room temperature for 15 min in 10 ml of distilled water. The previously made solutions were combined at room temperature in a 1:1 v/v% ratio, agitated for 20 min, and then ultrasonically agitated for 15 min [40].

### Loading of zinc oxide/chitosan nanocomposite with vancomycin

**Fig. 1:**
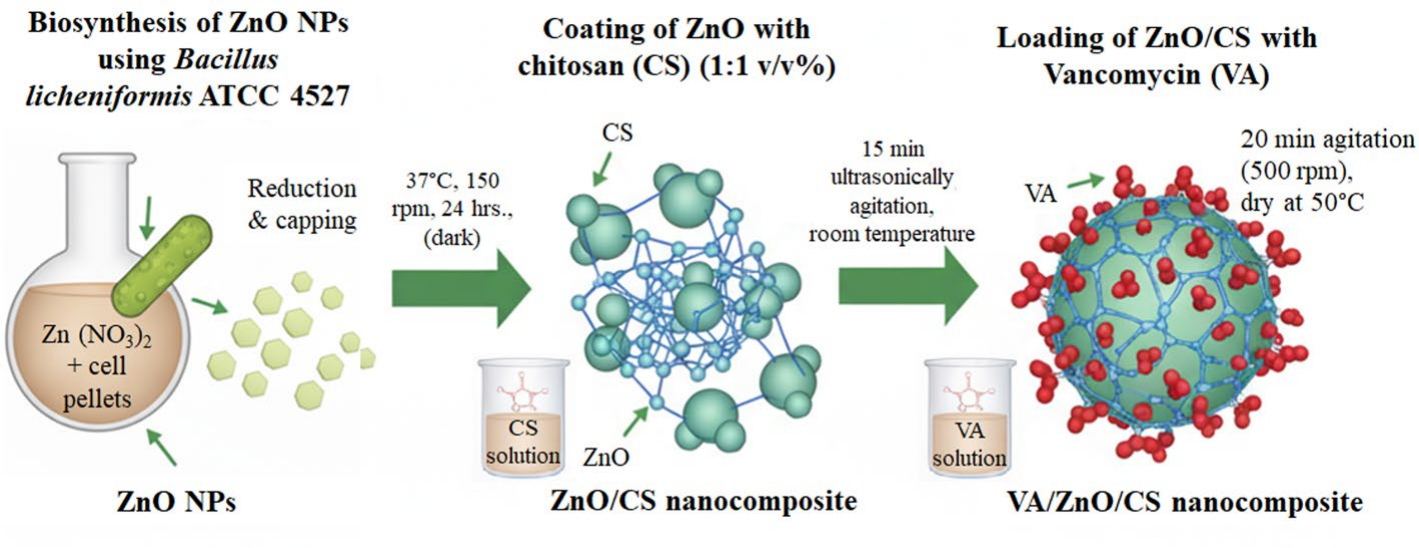
Sequential steps for the green synthesis of vancomycin-loaded ZnO/chitosan nanocomposite (VA/ZnO/CS)

A solution of zinc oxide/chitosan nanocomposite was mixed with 6 mg of the antibiotic VA at room temperature and agitated for 20 min at 500 rpm. The drug-loaded nanocomposite was then centrifuged at 5000 rpm after 6 h., and the residue was then rinsed three times with distilled water. The product was then dried for 24 h. at 50 °C. a schematic diagram illustrating the green synthesis of VA/ZnO/CS nanocomposite was displayed in Fig. 1.

### Characterization of VA/ZnO/CS

A double beam spectrum UV–Vis spectrophotometer V-630 (JASCO, UK) and Fourier transform infrared spectroscopy (FTIR, FT/IR-4100typeA) were utilized to analyze and study the VA/ZnO/CS. An X-ray diffractometer (model LabX XRD-6000, Shimadzu, Japan), a transmission electron microscope (TEM) apparatus (200 kV, TEM JEOL JEM-2100, Japan), and a zeta potential analyzer (Malvern Zetasizer Nano-ZS90, Malvern, UK) were employed to examine the properties of VA/ZnO/CS. The Scherrer equation (*D*_*hkl*_ = *kλ/β*_*hkl*_*cosθ*); *D* represents particle size, *K* is a constant equal to 0.94, *λ* is x-ray wavelength, *β* is full width at half maximum, _*hkl*_ are the Miller indices of the planes being analyzed, and *θ* is the angle of diffraction, was applied to calculate the average of crystallite size of NPs.

### Antimicrobial activity using agar well diffusion method

*Bacillus cereus*, MRSA, *P. mirabilis*, *K. pneumoniae*, and *C. albicans* were used as a Gram-positive bacteria, Gram-negative bacteria, and yeast models to assess the antimicrobial properties of ZnO NPs and VA/ZnO/CS [41, 42]. Nutrient agar and YEPA plates were prepared and inoculated with 1–2 × 10^8^ CFU/ml for bacteria or 1 × 10^6^ CFU/ml for yeast, respectively. Then, 100 µl of the prepared ZnO NPs, CS, and VA/ZnO/CS at a concentration of 150 µg/ml in sterile distilled water was added to wells (5 mm) in the nutrient agar plates. Fluconazole and vancomycin were used as common antibacterial and anticandidal agents, respectively. For 48 h., the agar plates were incubated at either 37 °C for bacteria or 28 °C for yeast. Zones of inhibition (ZOI) were measured and documented following the incubation period. Growth control, which including culture medium inoculated by the tested microorganism, and sterility control including culture medium and sterile distilled water were also tested to ensure the viability and robust growth of the microorganisms in the absence of any test material or drug. This was done and to ensure the sterility of the medium and the absence of any contaminating microbes or material-induced turbidity.

### Minimum inhibition concentration

The broth dilution method was utilized to determine the minimum inhibitory concentration (MIC) for ZnO NPs and VA/ZnO/CS [43, 44]. For 24 h, 100 µl of an overnight microbial culture was added to 50 ml of nutrient broth medium (for bacteria) or YEPB (for yeast) supplemented with varying concentrations (0–50 µg/ml) of ZnO NPs, CS, fluconazole, or VA/ZnO/CS. The cultures were then incubated for 24 h. at 150 rpm either at 37 °C for bacteria or 28 °C for yeast. MIC values (indicating no apparent growth) were obtained using UV–Vis spectrophotometry at 600 nm.

### Minimum microbicidal concentration

To determine the minimum microbicidal concentration (MMC), 10 µl of each MIC set was added to nutrient agar or YEPA plates. The plates were then incubated for 48 h. at either 37 °C for bacteria or 28 °C for yeast. Following the incubation period, the plates were inspected and MMC values were noted [45].

### Ultrastructural study

In YEPB broth medium, the *C. albicans* were exposed to VA/ZnO/CS (MMC value) for two hrs. at 28 °C. The cells were then washed and treated with 0.1 M cacodylate buffer (pH 7) and 2.5% glutaraldehyde before being sent to the Electron Microscope Unit at the Central Laboratory of Mansoura University in Egypt, for ultrastructure examination and monitoring. Following the removal of the fixative, the sample was fixed for 90 min using 2% osmium tetroxide and washed with 0.1 M buffer. A graded series of ethanol was used to dry the fixed cells. After drying, the cells were immersed in a 1:1 Epon-Araldite mixture for an hour. The mixture was then polymerized at 65 °C for 24 h. The cells were sectioned using an ultra-microtome (50 μm) and double-stained with uranyl acetate and lead citrate before being placed on carbon-coated copper grids (Type G 200, 3.05 μm diameter, TAAP, U.S.A.) using a TEM.

### Statistical analysis

The SPSS software, version 18, was used to statistically evaluate the data. As stated by O’Connor [46], each experiment’s data were presented using the mean ± standard deviation (SD) following one-way analysis of variance (ANOVA) and Duncan’s multiple range test. The significance level was set at *p* < 0.05.

## Results and discussion

### Intracellular biosynthesis of ZnO NPs

Biosystems aim to utilize microbes for various biotechnological purposes, such as producing nanomaterials. Many microorganisms, including bacteria, fungi, and algae, act as biocatalysts in biosystems to transform large quantities of matter into nanoscale compounds. Because of their rapid growth, small size, and ease of cultivation, bacteria are utilized as excellent nano-factories. They also enable the precise control of the size and shape of the NPs that were created [47]. Within 24 h, ZnO NPs were biosynthesized intracellularly by *B. licheniformis* ATCC 4527. The development of ZnO NPs was confirmed by a color shift and the formation of a white colloidal precipitate. The addition of Zn^2+^ caused the reaction’s color to change to whitish and hazy indicating that Zn^2+^ had been reduced to ZnO NPs. The solution appeared white and transparent when placed in distilled water [48]. ZnO NPs were extracted from bacterial pellets using ultrasonography after being cleaned at least three times with distilled water. The extracted and washed ZnO NPs precipitate was dried and calcined resulting in a yellowish white powder. The color change to yellowish white was due to the excitation of surface plasmon vibrations in the ZnO NPs [49].

### Synthesis and characterization of VA/ZnO/CS

**Fig. 2:**
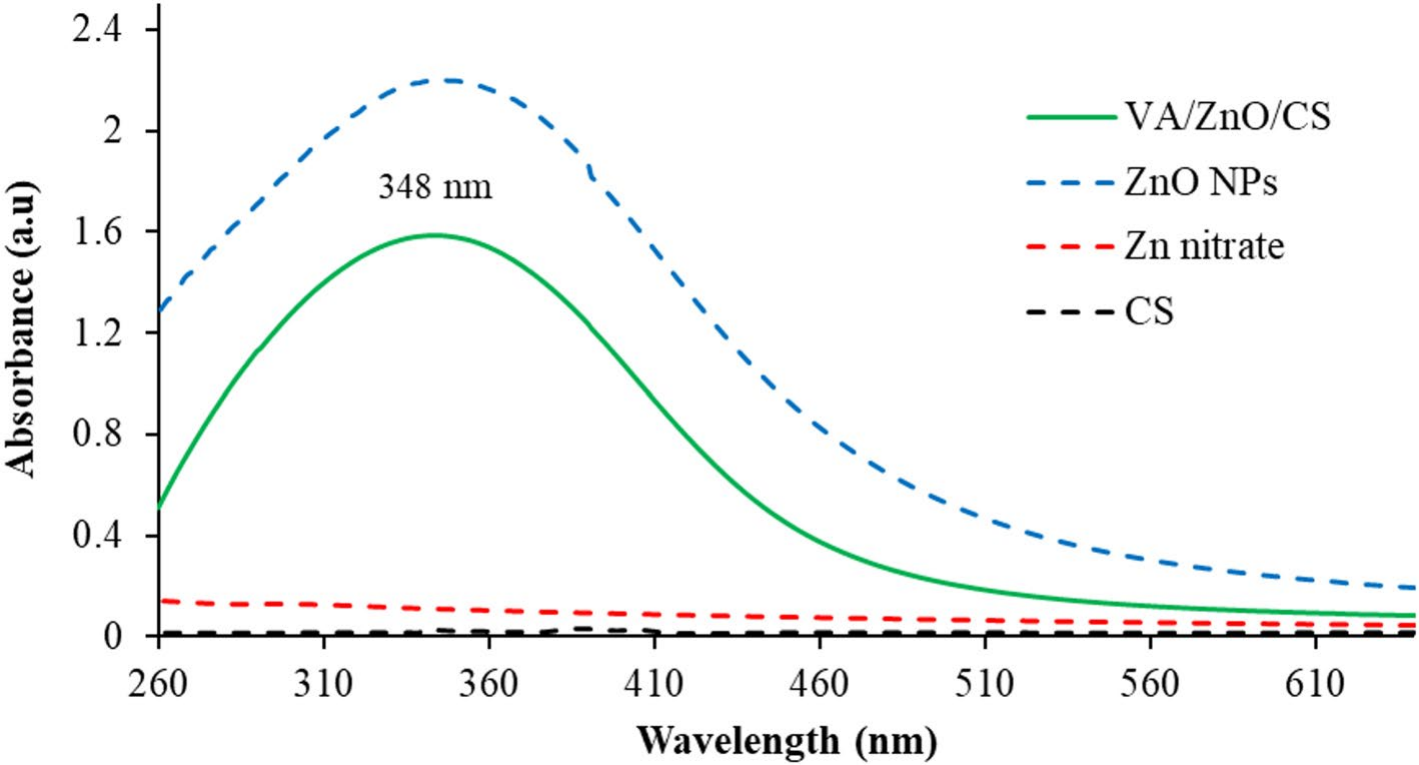
The UV–Vis spectra of Zn (NO_3_)_2_, ZnO NPs, and VA/ZnO/CS

UV–Vis spectroscopy analysis of VA/ZnO/CS revealed a distinct peak at 348 nm (Fig. 2). The reduction of Zn^2+^ to ZnO NPs was attributed to the presence of biological chemicals released into the supernatant by the bacteria and functional groups on the bacterial cell [48]. Using *B. subtilis* for 24 h, Ali et al. [50]. created a straightforward and affordable method for the biosynthesis of ZnO NPs, which showed an absorption peak at 375 nm. Furthermore, over the course of 24 h. at room temperature, Jayaseelan et al. [51]. used the reproducible bacteria *Aeromonas hydrophila* to biosynthesize ZnO NPs, revealing an absorbance peak at 374 nm. Salman et al. [52]. documented the biosynthesis of ZnO NPs at 80 °C over the course of 24 h utilizing *Lactobacillus* sp.

The UV–Vis absorption peak observed at 348 nm is not a deviation, but a fundamental spectroscopic signature that confirms the synthesis of small, highly active ZnO NPs, which are critical for the nanocomposite’s enhanced function. This peak represents a pronounced blue-shift when compared to the typical absorption of bulk ZnO (generally found around 370 nm) [53]. This blue-shift is directly explained by the Quantum Confinement effect, which dictates that as the size of a semiconductor crystallite decreases below its excitonic Bohr radius, the band gap energy increases, forcing the material to absorb light at shorter wavelengths [54]. Therefore, the 348 nm reading is a robust indicator that the ZnO NPs prepared were successfully formed with a small crystal size, maximizing their specific surface area and reactivity for generating ROS [55]. Furthermore, the successful surface functionalization by the CS polymer stabilizes the ZnO core and minimizes surface defects that could cause absorption in the visible range, ensuring the high quality and purity of the final VA/ZnO/CS [56].

**Fig. 3:**
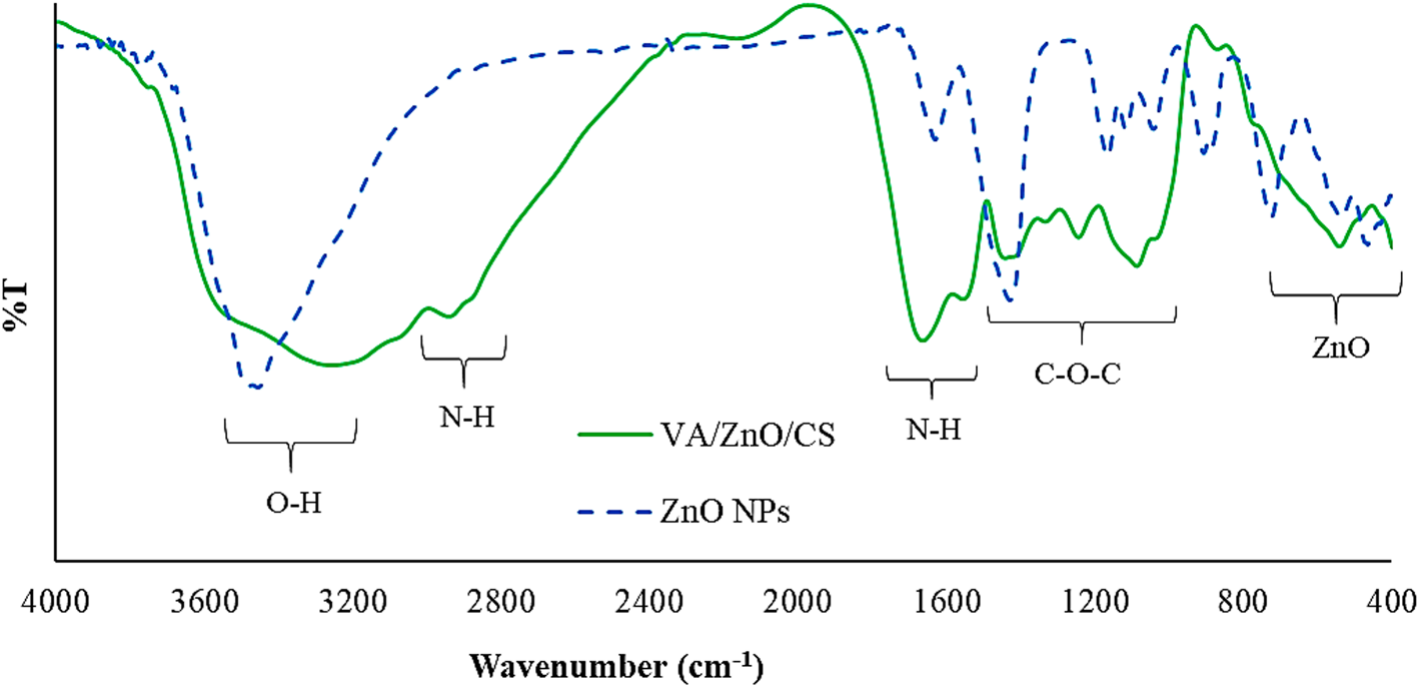
FTIR spectra of ZnO NPs and VA/ZnO/CS

Hydroxyl (O–H) groups are indicated by the prominent broad peaks at 3458 cm^− 1^ in the FTIR spectrum of ZnO NPs (Fig. 3). Bands at 1628 cm^− 1^ are associated with proteins in the amide I (N–C=O-stretching mode). Bands at 1200 –1030 cm^− 1^ represent amide II (the N–H bending mode) and amide III. Additionally, the bands at 1428 cm^− 1^ may be attributed to the C–N stretching mode. The presence of amines as a capping agent was validated by the VA/ZnO/CS FT-IR spectrum, with stretching peaks observed at 1553–1665 cm^− 1^ for primary amines and 2941 cm^− 1^ for asymmetric amines. The accumulation and aggregation of NPs are inhibited by the presence of carbonyl groups from amino acid residues and certain proteins, which have a significant ability to bind metal from metal NPs, such as capping ZnO NPs residues [57]. Esters and carboxylic acids are observed at 1075–1335 cm^− 1^, while hydroxyl groups are seen at 3258 cm^− 1^. The successful loading of ZnO with ZnO/CS nanocomposite is confirmed by the vibration stretching band of ZnO identified at multiple peaks ranging from 460 to 800 cm^− 1^. These findings align with the FT-IR results and explanations provided in several previous studies [58–62].

**Fig. 4:**
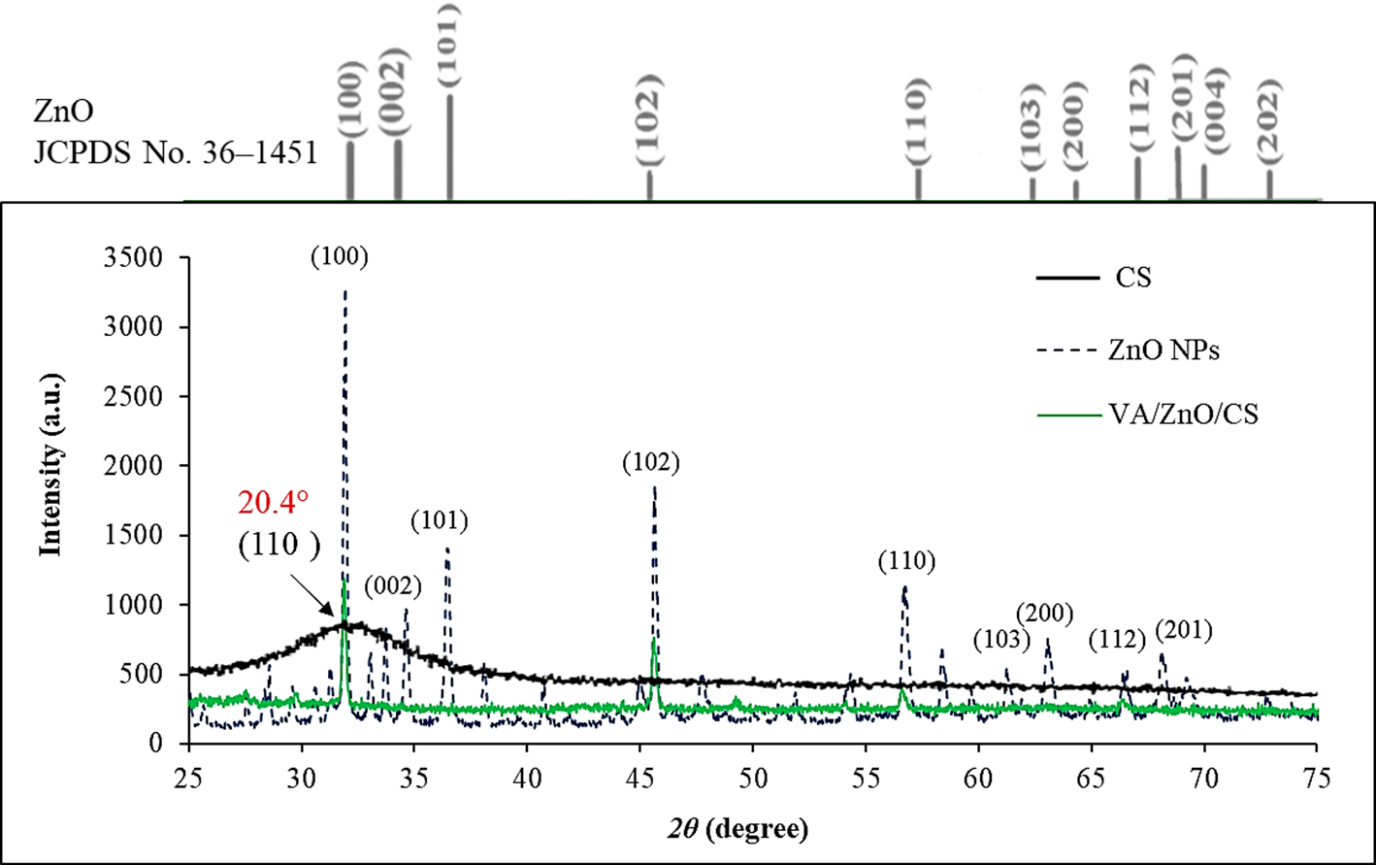
XRD patterns of Cs, ZnO NPs, and VA/ZnO/CS

The XRD spectra of ZnO NPs and VA/ZnO/CS displayed the purity and well-crystalline nature of the prepared nanocomposite (Fig. 4). The selecting for calcination temperature of 500 °C for the ZnO NPs hinges on optimizing the critical trade-off between crystallinity, purity, and active surface area, all of which govern the material’s antimicrobial efficiency [63]. This temperature ensures the complete thermal decomposition of precursor materials into pure ZnO in its highly stable wurtzite crystalline phase, which is essential for product consistency. Critically, 500 °C is high enough to achieve good crystallinity and purity, but low enough to inhibit excessive sintering and crystal growth that occurs at higher temperatures. By minimizing this particle growth, the NPs retain a high specific surface area and a suitable defect density, maximizing the available active sites for bacterial contact, efficient Zn^2+^ ion release, and the generation of ROS [55]. Therefore, 500 °C represents the optimal condition to ensure the ZnO component possesses the necessary physicochemical characteristics for maximum antimicrobial contribution within the synergistic VA/ZnO/CS nanocomposite.

Sharp peaks at 31.91°, 34.62°, 56.67°, 66.39°, and 75.44° correspond to the lattice planes (100), (102), (110), (200), and (202), respectively, confirming the integration of ZnO NPs in VA/ZnO/CS. The peaks of ZnO NPs agreed with the Joint Committee of Powder Diffraction Standards (JCPDS) card No. 36-1451 [64]. The distinct broad, semi-crystalline peak centered at 20.4° is clearly visible in the pure CS pattern (black line, Fig. 4), consistent with previous research [65, 66]. Between 19° and 21°, a prominent, broad peak emerges. This peak is often attributed to the (110) crystal planes of the CS unit cell in the Form II crystal structure making it the most distinctive feature of the crystalline structure of CS. The high degree of deacetylation (≥ 85%) promotes greater regularity in the polymer chains strengthening intramolecular and intermolecular hydrogen bonds and resulting in more pronounced crystalline peak. The addition of ZnO NPs leads to significant changes in this area in the resulting VA/ZnO/CS material. The main distinctive peak of CS has been shifted to lower angle (approximately 20.1°) and has also widened. This alteration, coupled with a noticeable decrease in peak intensity, indicates a reduction in the overall crystallinity of the CS matrix. The likely reason for this phenomenon in the strong intermolecular interactions such as hydrogen bonds, between the ZnO particles polymer chains due to the disruptive intercalation of the ZnO NPs. The structure of nanocomposite becomes less ordered and more amorphous as a result of these interactions, which prevent the CS chains from packing neatly [67]. Since no additional impurity-corresponding diffraction peaks are detected, the high purity of the produced goods is confirmed [68]. The crystalline size of NPs was calculated and indicating a mean crystalline size of 79.38 nm according the Scherrer equation.

**Fig. 5:**
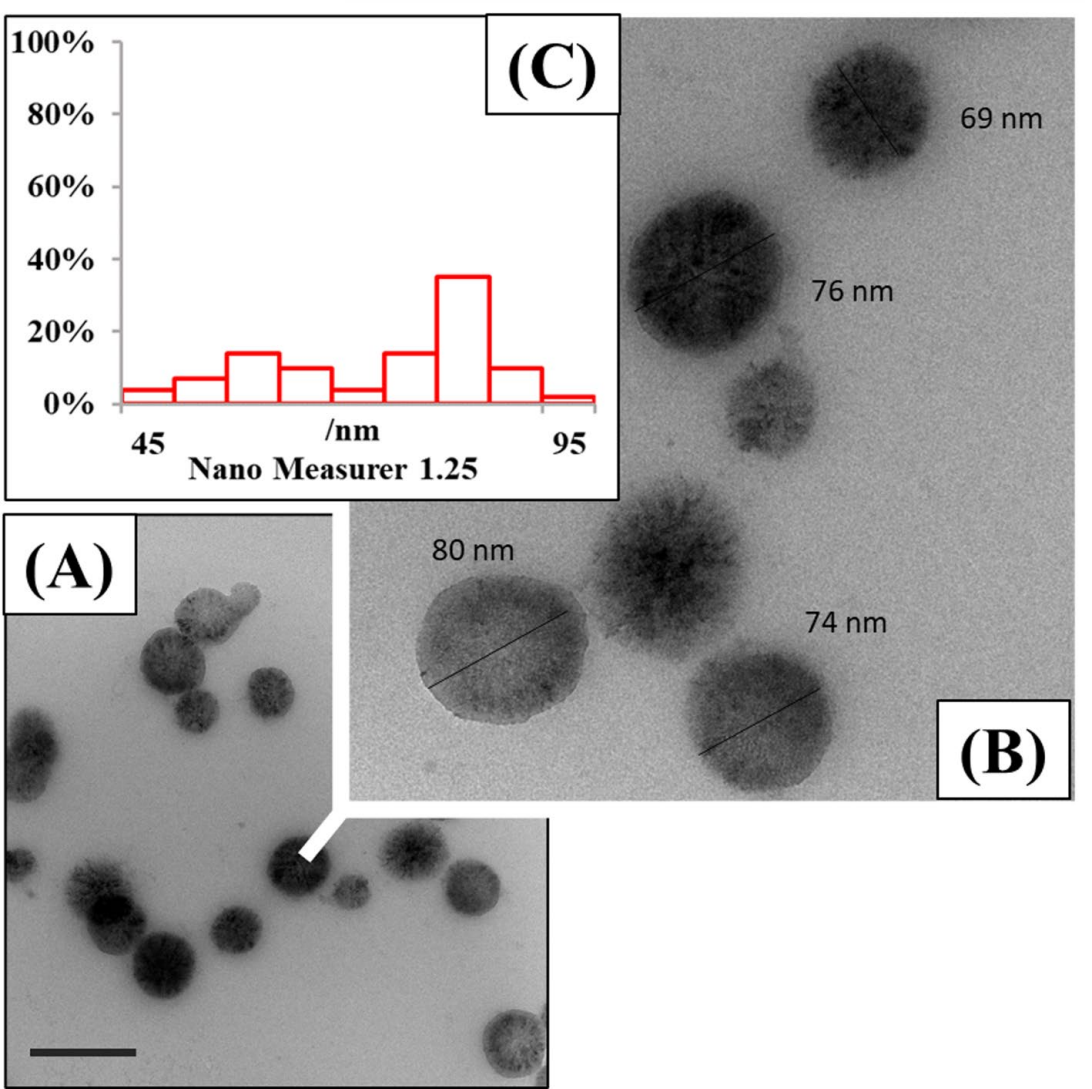
**A** TEM of VA/ZnO/CS with scale bar = 100 nm. **B** Magnified part showed the spherical shaped ZnO NPs with size particle range 45–95 nm. **C** Nanogravimetric image showing the particle size distribution

The TEM is an essential instrument for determining the dimensions and shape of the produced NPs. Figure 5 shows the TEM micrograph of VA/ZnO/CS. The average diameter of the spherical NPs was calculated using using Nano Measurer 1.25 software and recorded as 78 ± 2.3 nm. The generated NPs appear to be single-domain crystals and exhibit a high degree of crystallinity. For this particular material system, the expected crystallite size as determined by the Scherrer equation should thus be reasonably indicative of the actual particle size. The average particle diameter of 78 ± 2.3 nm as determined by TEM is in excellent agreement with the mean crystallite size of 79.38 nm as determined by the Scherrer equation. By condensing DNA molecules, inhibiting replication, deactivating enzymes, and disrupting metabolism, smaller NPs can damage microbial protoplasts and have a higher antibacterial effect than larger ones [69, 70]. These outcomes demonstrate the effectiveness of the process used in this work to produce pure ZnO NPs. Furthermore, the data show that the diffraction peaks in the ZnO NPs produced had a superior crystalline structure as the annealing temperature increased, becoming smaller and more intense [71]. The average size of ZnO/CS was found to be 338.7 nm by Mehta et al. [72], which contrasts with the current experiment. Rehman et al. [73] reported that the size of ZnO NPs biosynthesized by *B. haynesii* varied between 50 and 55 nm. However, according to Yusof et al. [48], the diameter of ZnO NPs produced by *L. plantarum* ranged from 90 to 124 nm.

**Fig. 6:**
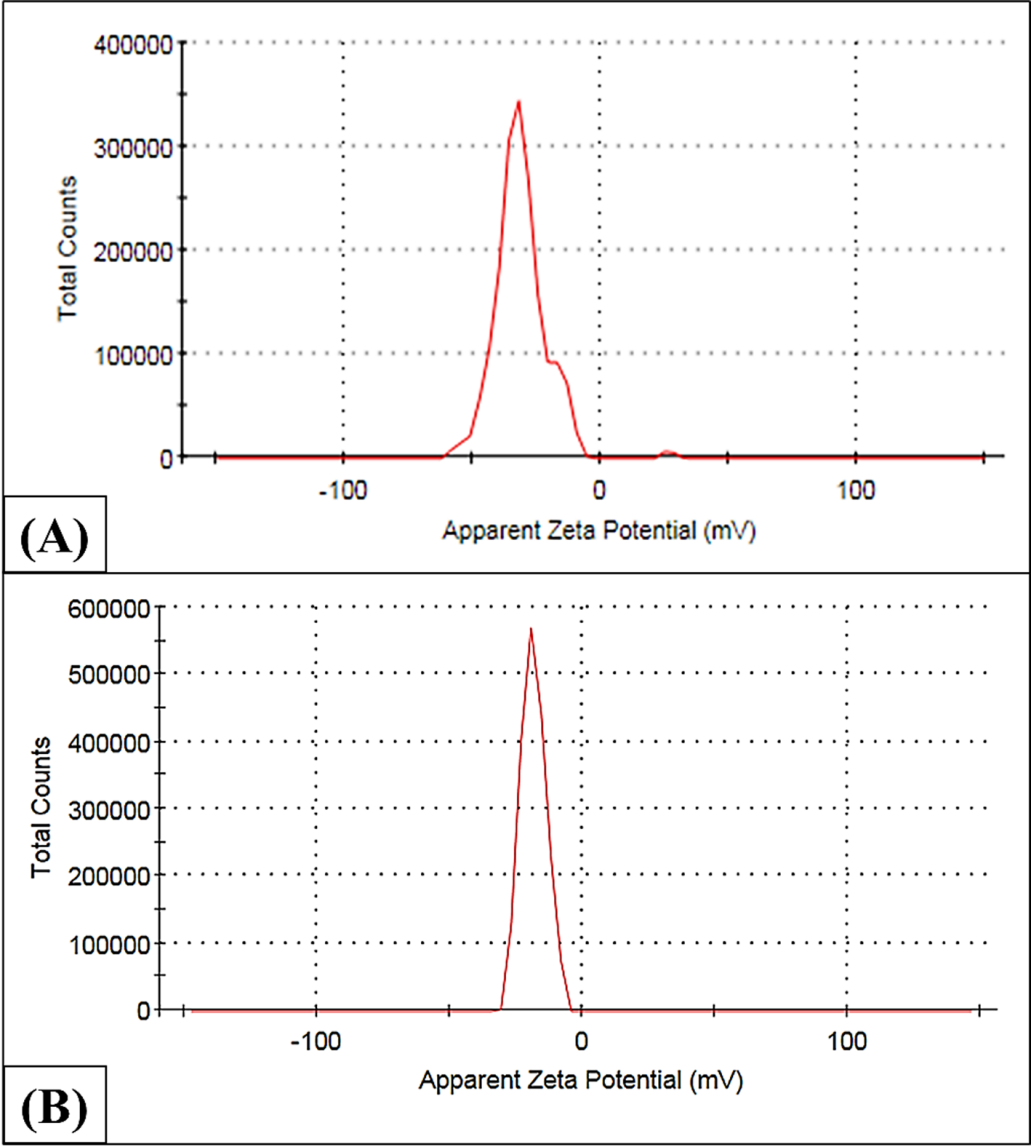
**A** Zeta potential of ZnO NPs. **B** Zeta potential of VA/ZnO/CS

The magnitude of the zeta potential value indicated that the NPs and nanocomposite were stable. As shown in Fig. 6, the zeta potential data revealed the negative charge of the green synthesized nanocomposite (− 28.2 mV for ZnO NPs and − 17.8 mV for VA/ZnO/CS). The significant negative charge of the zeta potential prevents NPs from clustering together, thus enhancing their stability [74]. It is clear from the generated NPs that the particles are relatively stable. According to Iqtedar et al. [75], NPs’ zeta potential is considered almost neutral when it is between − 10 and + 10 mV. However, a study by Abdelhakim et al. [76] showed that ZnO NPs were more stable, with a negative zeta potential of − 23.92 mV. Considering the usual positive charge of free CS (due to its protonated amine groups, NH_3_^+^), It is unexpected that the final VA/ZnO/CS composite has a negative zeta potential of − 17.8 mV. Two primary reasons were attributed to this outcome as reported by Antony et al. [77]. First, the biosynthesized ZnO NPs have a very significant initial negative charge of − 28.2 mV. Different anionic components (such as proteins, polysaccharides, and other biomolecules) inherited from the bacterial bioactive compounds and bio-reduction create and stabilize the ZnO NPs. Secondly, the strongly negative surface of the ZnO NPs interacts with the positively charged CS through electrostatic forces. The unshielded negative charge of the ZnO NPs surface still determines the final complex’s overall surface potential in this interaction, even if the CS partially envelops the NPs. Alternatively, the strong anionic species surrounding the ZnO NPs may complex or shield the CS-amine groups, leaving a residual negative potential for the entire nanocomposite. The total surface charge has been slightly neutralized by the positive CS, but not sufficiently so to change it to a positive value, as evidenced by the measured value of − 17.8 mV being less negative than the free ZnO NPs (− 28.2 mV).

### Antimicrobial activity

The prepared NPs and nanocomposite demonstrated potent antibacterial activity against every tested pathogen, as determined by the agar well diffusion test (Table 1). When compared to the conventional drugs vancomycin and fluconazole, which induced inhibition zones of 16 ± 0.14 mm to 21 ± 0.03 mm against bacteria and 18 ± 0.03 mm against yeast, VA/ZnO/CS demonstrated higher antibacterial and anticandidal action against Gram-positive, Gram-negative bacteria and *C. albicans* with inhibition zones of 16 ± 0.06 mm to 25 ± 0.03 mm and 31 ± 0 mm, respectively.

Additionally, it was shown that ZnO NPs had a modest anticandidal effect of 9 ± 0.14 mm and that *C. albicans* exhibited complete resistance activity against CS (no inhibition zone). The results obtained by Kyu Aung and Thida-Htun [78] showed that a chemically synthesized ZnO/CS nanocomposite using the co-precipitation method recorded inhibition zones of 10–14 mm, 15–19 mm, 20 mm, and 30 mm against *P. mirabilis*, MRSA, *B. cereus*, and *C. albicans*, respectively. The study also reported that there was no inhibition zone for CS against the same strains. In a study by Ali et al. [79], it was reported that chemically formulated ZnO/CS and physically prepared ZnO/CS nanocomposites exhibited antibacterial and anticandidal properties, with inhibition zones ranging from 12 to 15 mm and 15–21 mm against *E. coli*, *S. aureus*, *B. cereus*, and *C. albicans*. According to Yassin et al. [80], *C. albicans* has shown complete resistance to fluconazole. However, our current study found that fluconazole had a moderate anticandidal effect against *C. albicans*, but it also had negative health effects such as hepatotoxicity and hormone-related side effects like alopecia, oligospermia, azoospermia, hypokalemia, and hyponatremia [81].

**Table 1:** Agar well diffusion method of ZnO NPs, CS, and VA/ZnO/CS compared to the standard drugs

Antimicrobial agents	Zone of inhibition (mm, mean ± SD)				
	Gram-positive bacteria		Gram-negative bacteria		Yeast
	*B. cereus*	MRSA	*P. mirabilis*	*K. pneumoniae*	*C. albicans*
Vancomycin	18 ± 0.03	21 ± 0.03	16 ± 0.14	−ve	–
Fluconazole	–	–	–	–	18 ± 0.03
ZnO NPs	20 ± 0.03	23 ± 0.03	17 ± 0.14	12 ± 0.14	9 ± 0.14
CS	−ve	−ve	−ve	−ve	−ve
VA/ZnO/CS	23 ± 0.06	25 ± 0.03	20 ± 0.06	16 ± 0.06	31 ± 0

The VA/ZnO/CS nanocomposite offers substantial benefits in tackling AMR by overcoming the limitations of conventional antibiotic monotherapy, including individual antibiotics or NPs, through synergistic action, enhanced delivery, and broad-spectrum efficacy. Table 2 summarizes the key features of various common commercial NPs and highlights how VA/ZnO/CS addresses their limitations. The current combination is designed to make resistance development highly improbable: VA targets the D-Ala–D-Ala termini in Gram-positive cell walls, while the ZnO NPs provides a simultaneous, non-specific physical-chemical attack by generating ROS and releasing Zn^2+^ ions that damage membranes and disrupt metabolic pathways [82, 83]. This synergy—where ZnO likely permeabilizes the membrane to enhance VA’s access to its target—allows for lower VA concentrations, significantly reducing the selective pressure that drives the evolution of drug-resistant strains like *K. pneumoniae*. Furthermore, the CS biopolymer acts as a targeted delivery vehicle due to its positive charge binding to bacterial surfaces, and, critically, it possesses potent anti-biofilm activity to break down the protective matrices that shelter bacteria from conventional drugs [84, 85]. This enhanced local delivery and superior biocompatibility also support in vivo applications (e.g., medical coatings or wound dressings) with reduced toxicity compared to systemic VA. Finally, the inclusion of ZnO extends the product’s utility by providing collateral activity against certain Gram-negative pathogens, offering a dual-mechanism solution for treating complex, mixed-species infections.

**Table 2:** Comparative analysis of commercial NPs versus VA/ZnO/CS nanocomposite

NPs/ nanocomposite	Primary antimicrobial mechanism	Key strength in commercial use	Primary limitation	VA/ZnO/CS advantages
Silver (Ag)	Release of Ag^+^ ions; non-specific binding to bacterial DNA/proteins [86]	Potent broad-spectrum activity (Gram-positive and Gram-negative)	Cytotoxicity at effective doses; high cost; potential for environmental pollution	Reduced toxicity: Uses CS to control ZnO release, allowing for lower toxic component concentration while maintaining efficacy *via* synergy
Copper (Cu)	Cu^2+^ ion release; membrane damage; ROS generation [87,88]	Lower cost than Ag; strong contact killing (surface coatings)	High Cu^2+^ ion release can lead to cytotoxicity and genotoxicity; poor stability (oxidizes easily)	Stability and control: CS matrix enhances stability and allows for controlled, sustained release of the active agents, minimizing burst toxicity
ZnO	ROS generation; Zn^2+^ ion release; membrane disruption [55]	Low cost; good UV-blocking properties; generally recognized as safe (GRAS)	Limited intrinsic efficacy against many highly resistant pathogens (MRSA); primary mechanism is non-specific (easy for resistance evolution)	Synergy with VA: VA provides the specific targeting (Gram-positive) that ZnO lacks, achieving vastly superior efficacy with a lower dose
Titanium dioxide (TiO_2_​)	Strong ROS generation under UV irradiation (photocatalytic) [89]	Highly stable; inexpensive; excellent surface disinfectant	Requires UV light activation (not practical for *in vivo* applications or opaque materials); low efficacy in dark conditions	Efficacy is light-independent, relying on chemical synergy and inherent material properties (VA, CS), making it ideal for *in vivo* applications
ZnO/TiO_2​_	Combination of ZnO and TiO_2_​ mechanisms [90]	Enhanced photocatalytic activity and better charge separation than single oxides	Still heavily reliant on UV light for maximum effect; still lacks specific antibiotic targeting	Incorporates VA for specific targeting of clinically relevant strains (MRSA), moving beyond non-specific oxidative stress
VA/ZnO/CS	VA target specificity + ZnO ROS + CS anti-microbial/targeting	Multi-mechanism synergy that addresses two major AMR challenges: resistant strains (VA/ZnO synergy) and targeting (CS effect)	Novelty means higher initial development cost and regulatory hurdles	Superior efficacy and low resistance potential. The triple-component mechanism is a “hard target” for bacteria to develop resistance against compared to single-mechanism commercial alternatives

**Fig. 7:**
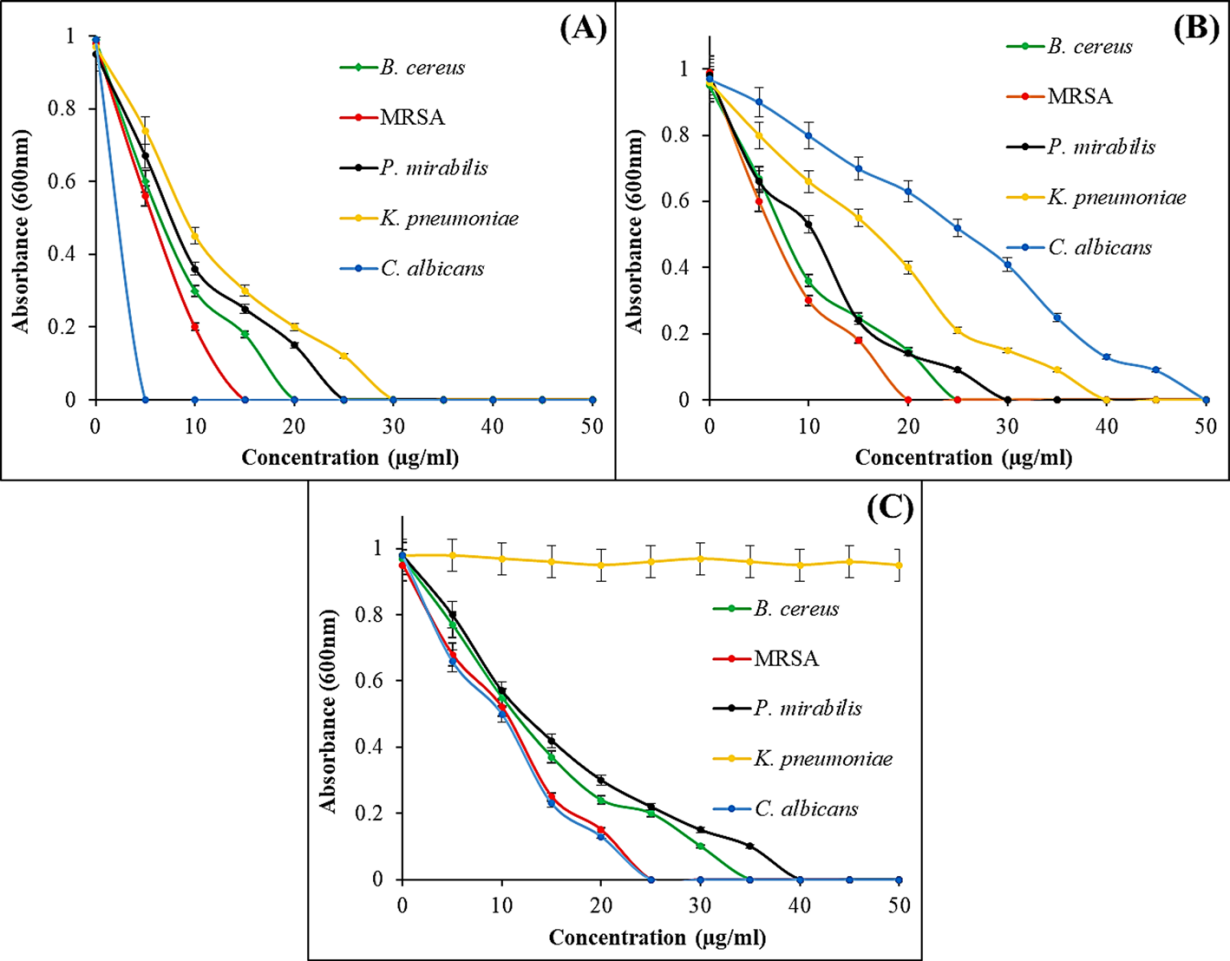
Minimum inhibition concentration of VA/ZnO/CS; (**A**), compared to ZnO NPs; (**B**), and standard drugs; (**C**) against the tested microbial strains

**Fig. 8:**
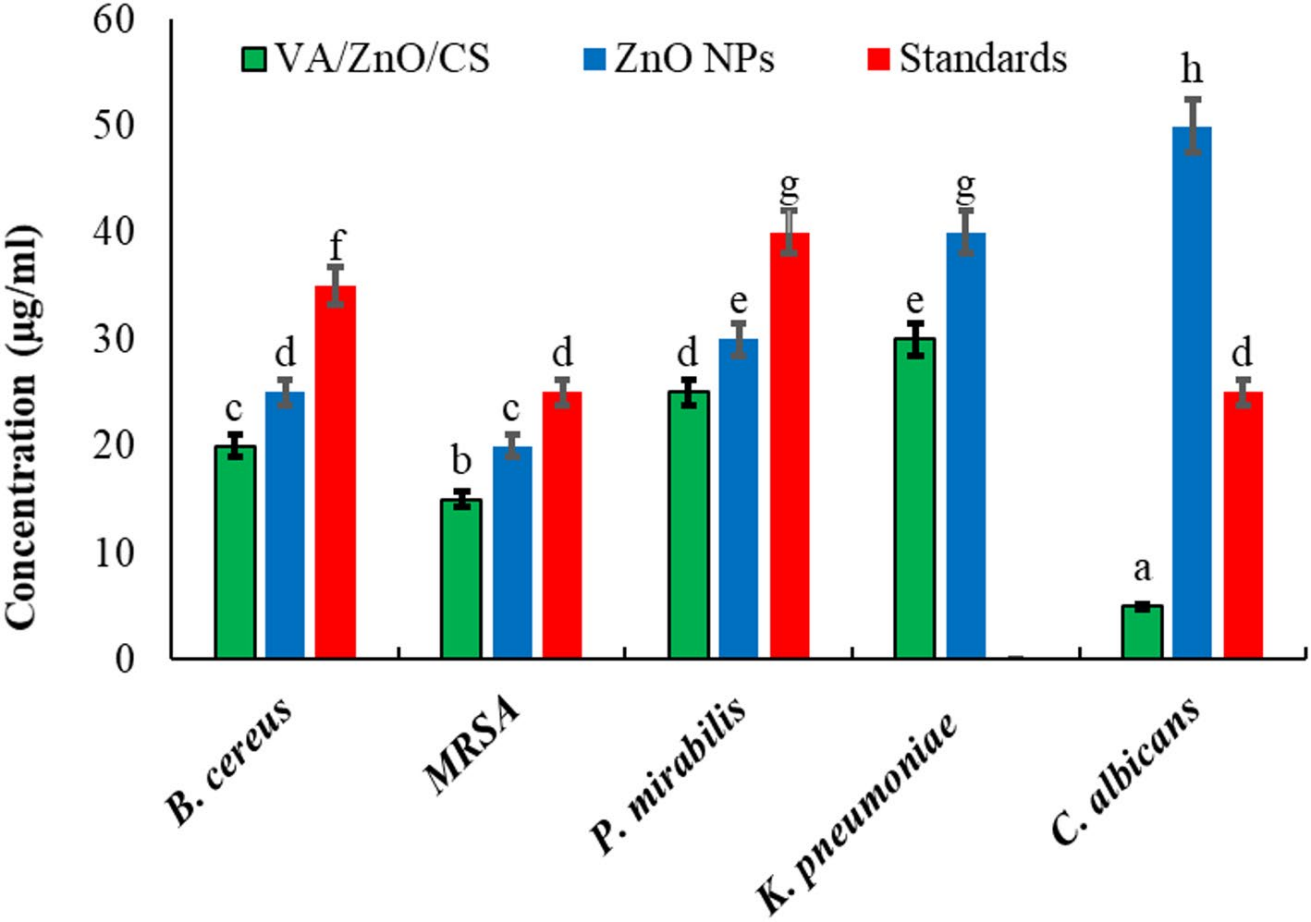
Minimum microbicidal concentration of VA/ZnO/CS compared to ZnO NPs and standard drugs against the tested microbial strains

The antimicrobial efficacy of the synthesized compounds was quantitatively assessed by determining the MIC against a panel of five critical strains, as illustrated in Fig. 7; Table 3. The most striking observation from the MIC data is the exceptionally high antifungal potency of the VA/ZnO/CS against *C. albicans*. VA/ZnO/CS exhibited an estimated MIC of approximately 5 µg/ml against *C. albicans*. It was characterized by a steep dose-response curve that reached complete growth inhibition at the lowest concentration among all tested strains and compounds (Fig. 7A). In sharp contrast, the unmodified ZnO NPs) (Fig. 7B) demonstrated poor antifungal activity. The growth curve remained near maximum absorbance even at 50 µg/ml, suggesting an MIC value exceeding the tested concentration range (>50 µg/ml). This demonstrates a >10-fold enhancement in antifungal activity against *C. albicans* through the VA/ZnO/CS formulation compared to bare ZnO NPs. This finding is critical, as it suggests that the nanocomposite effectively addresses a major clinical challenge: fluconazole-resistant candidiasis, by overcoming the inherent limitations of both traditional NPs and conventional antifungal agents. The synergistic action of VA and CS, in combination with the Zn^2+^ release mechanism, likely facilitates superior membrane interaction and permeability, driving the low MIC. The antifungal activity of ZnO NPs and ZnO/CS nanocomposite against *C. albicans* was reported by Dananjaya et al. [91], with MIC values of 200 µg/ml and 75 µg/ml, respectively. Wang et al. [92] reported that the MIC of the ZnO/CS nanocomposite against *C. albicans* was 160 µg/ml. Furthermore, the nanocomposite demonstrated a broad-spectrum effect, showing superior or comparable activity against both Gram-positive and Gram-negative bacteria. The consistent decrease in MIC values for VA/ZnO/CS compared to ZnO NPs across all bacterial strains confirms that the bi opolymer functionalization strategy enhances antimicrobial performance. The observed difference in MIC between Gram-positive and Gram-negative strains (e.g., 20 µg/ml for *B. cereus* vs. 35 µg/ml for *K. pneumoniae*) is in line with existing literature, highlighting the protective role of the Gram-negative outer membrane. However, the effectiveness against highly resistant *K. pneumoniae* is particularly and suggests potential in addressing urgent multi-drug-resistant pathogens. The standard drug treatment (Fig. 7C) was completely ineffective against *K. pneumoniae*, showing no reduction in absorbance across the entire concentration range (0–50 µg/ml). It was noted that the recorded the MICs of VA/ZnO/CS were matched with its MMCs indicating the potent biocidal action of the prepared nanocomposite against the tested microorganisms (Fig. 8). Mahmoud et al. [93] reported the antibacterial activity of the ZnO NPs with an MIC of 2.5 µg/ml against *E. coli* and 1.25 µg/ml against *S. aureus*. The study also recorded the MBC of ZnO NPs as 2.5 µg/ml against the same strains. While El-Khawaga et al. [94] documented the MICs of ZnO NPs against *S. aureus* and *E. coli* as 0.625 µg/ml and 1.250 µg/ml, respectively. Ali et al. [79] was also prepared ZnO/CS and tested its antibacterial activity against *S. aureus*, *B. subtilis*, *E. coli*, and *K. pneumoniae*. The MIC and MBC values fell in the range of 30–250 µg/ml.

**Table 3:** Minimum inhibitory concentration of VA/ZnO/CS compared to ZnO NPs against selected microbial strains

Strain	VA/ZnO/CS MIC (µg/ml)	ZnO NPs MIC (µg/ml)	Comparative analysis
*Bacillus cereus* (Gram-positive bacteria)	20	25	20% improvement over ZnO NPs, excellent Gram-positive antibacterial activity, and better than standard
MRSA (Gram-positive bacteria)	15	20	25% improvement over ZnO NPs, highest antibacterial potency, and comparable to the standard drug (25 µg/ml)
*Proteus mirabilis* (Gram-negative bacteria)	25	30	16.7% improvement over ZnO NPs, good antibacterial action against Gram-negative bacteria, showing clear enhancement
*Klebsiella pneumoniae* (Gram-negative bacteria)	30	40	25% improvement over ZnO NPs, highly effective against the challenging strain
*Candida albicans* (yeast)	5	50	900% improvement over ZnO NPs, exceptional antifungal potency, and 5-fold better than fluconazole

**Fig. 9:**
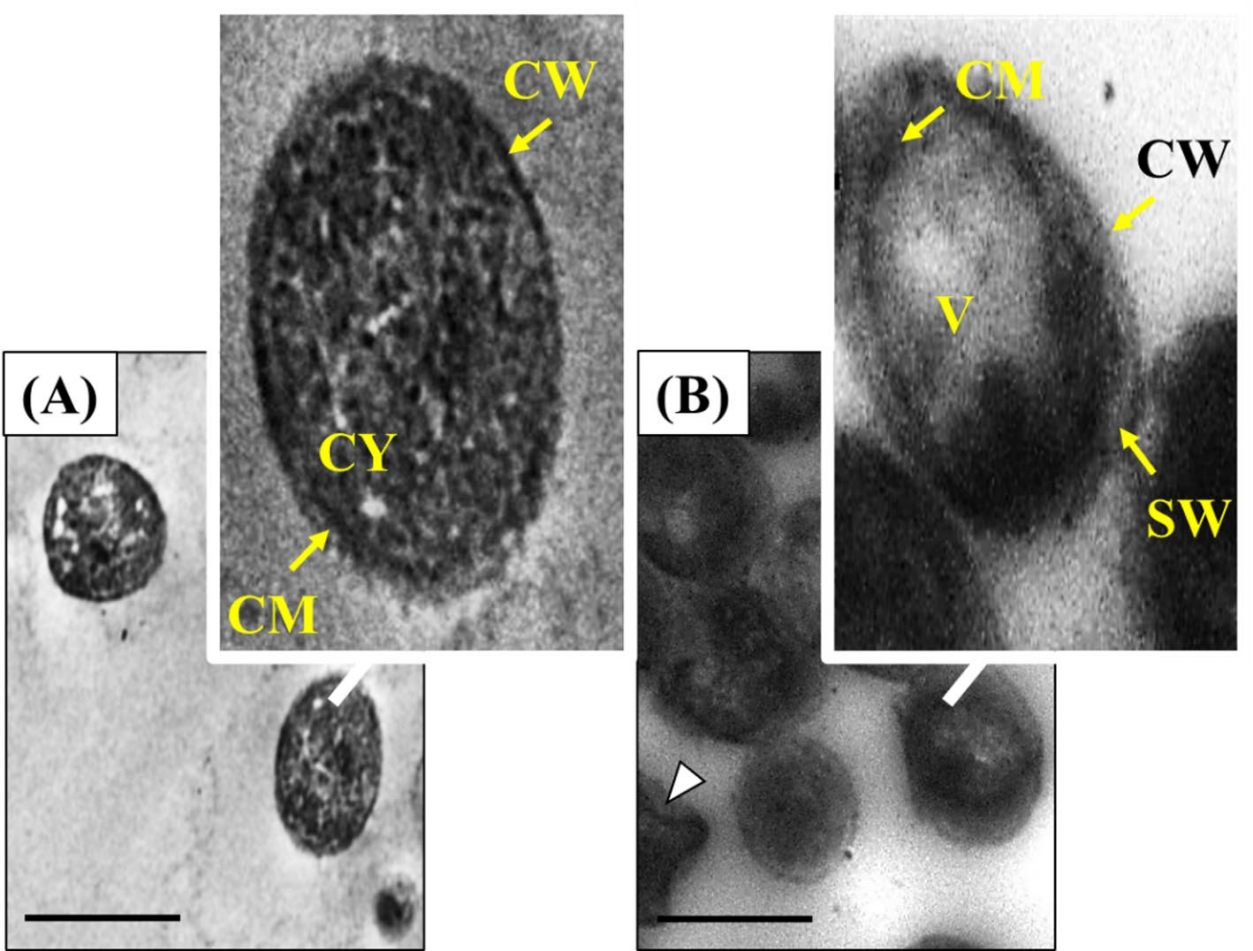
Anticandidal action of VA/ZnO/CS on the ultrastructure of *C. albicans* cells (**B**), comparing to untreated cells (**A**). CW is cell wall. CM is cytoplasmic membrane. CY is cytoplasm. SW refers to a separation between the cell wall and the cytoplasmic membrane. V is a vacuole. White arrowheads indicated the presence of malformed cells

The TEM images in Fig. 9 provide direct visual evidence of the mechanistic action behind the VA/ZnO CS Nanocomposite’s exceptional antifungal activity against *C. albicans*. Figure 9A, represents the untreated control cell, displaying a pristine and intact cellular architecture, with clearly defined, tightly associated layers: the cell wall, the underlying cytoplasmic membrane, a dense cytoplasm, tiny vacuoles, and normal cell membranes. In stark contrast, Fig. 9B, which shows the VA/ZnO/CS-treated cells, reveals severe ultrastructural damage consistent with rapid fungicidal action. The most critical observed feature is the separation between the cell wall and the cytoplasmic membrane, indicating a complete compromise of the protective cell envelope and loss of structural integrity. Furthermore, the treated cells exhibit large, irregular vacuoles and appear malformed, signifying internal organelle damage, cellular stress, and total loss of homeostasis. This visual evidence directly correlates with the strong numerical results, where the VA/ZnO/CS achieved an MMC of just 5 µg/ml, confirming that the VA/ZnO/CS efficiently kills the fungus by physically and chemically disrupting its cell wall and membrane, leading to irreversible internal cytoplasmic collapse. Scientists have suggested several possible microbicidal actions of zinc oxide, but the exact processes underlying its antimicrobial action remain unknown. One of these actions is the release of Zn ions, which is associated with the accumulation of NPs in microbial cells [95]. ZnO NPs interact electrostatically with cell walls, leading to the production of ROS and the destruction of microbial cell integrity [96]. Smaller NPs may have an easier time entering microbial cells, allowing them to generate more ROS, which can induce cell death, denaturation of proteins, and damage to cells [66]. Figure 9 provides direct visual confirmation that the VA/ZnO/CS exerts its anticandidal effect through a multi-pronged attack on the fungal cell. The current study state that the mechanism of the anticandidal action of the prepared nanocomposite is primarily characterized by the disruption of the cell envelope, specifically the separation of the cell wall from the cytoplasmic membrane, which leads to osmotic imbalance, severe vacuolization, and irreversible internal organelle damage. The presence of vacuolization inside the cytoplasm, indicates severe damage to intracellular organelles and cellular stress. This internal damage has led to osmotic stress and disruption of metabolic processes caused by release of Zn ions, which may result in the generation of ROS. The overall shape of the cell is malformed, and the cytoplasm appears less dense and aggregated. There has been a total loss of homeostasis, leading to the leakage of essential intracellular contents and eventual cell death.

The VA/ZnO/CS nanocomposite was specifically engineered to leverage the synergistic antimicrobial mechanisms of its components while maintaining superior biocompatibility and reduced toxicity compared to high-dose systemic therapy. The nanocomposite achieves high antimicrobial efficiency through a multi-pronged, synergistic, and broad-spectrum attack that is exceptionally difficult for bacteria to overcome. Crucially, the synergy between ZnO NPs and VA—where ZnO likely disrupts the cell membrane—allows for enhanced VA penetration and consequently permits the use of a lower effective VA dose, thereby mitigating the selective pressure that drives resistance [97]. Furthermore, the cationic CS component is vital for both targeting delivery and its potent anti-microbial effects, actively preventing and disrupting the highly resistant structures implicated in chronic AMR infections [98]. This comprehensive mechanism of action expands the composite’s utility, with the ZnO component offering collateral activity against certain Gram-negative pathogens. The safety and biocompatibility of the VA/ZnO/CS nanocomposite are primarily established through its materials and its dose-sparing strategy. CS is a well-established, biodegradable, and non-toxic polymer that serves as an excellent, biocompatible scaffold [99]. The composite’s synergistic efficiency allows for a dramatic reduction in the required effective VA concentration, directly addressing the major safety concern of VA—nephrotoxicity—associated with systemic, high-dose administration. This localized treatment approach is anticipated to significantly reduce systemic exposure and associated toxicity [100]. Moreover, the CS matrix ensures the stable and controlled release of VA and Zn^2+^ ions, preventing toxic burst release [101]. Importantly, in vitro toxicity studies against mammalian cells at the MICs are required to investigate the cytotoxicity of the prepared nanocomposite, thereby demonstrating a favorable therapeutic window for clinical and pharmaceutical applications.

## Conclusions

This study successfully developed a novel VA/ZnO/CS using a simple green synthesis approach, with *B. licheniformis* serving as both a stabilizer and a reducing agent. Comprehensive structural characterization confirmed the formation of the nanocomposite: UV–Vis spectroscopy showed a peak at 348 nm, XRD identified key ZnO diffraction peaks (e.g., 31.77°), FT-IR confirmed ZnO stretching vibrations (at 460–800 cm^− 1^), Zeta potential analysis measured a charge of − 17.8 mV, and TEM revealed spherical-shaped NPs with an average diameter of 78 ± 2.3 nm. Crucially, the VA/ZnO/CS was established as the most potent agent across the entire tested panel of bacterial and fungal strains. Its unique formulation successfully lowered the MIC profile for all strains, particularly demonstrating dramatically improved antifungal performance where the precursor material and commercial agents struggled. Specifically, the green-synthesized VA/ZnO/CS exhibited potent anticandidal activity against *C. albicans*, with both MIC and MFC values of 5 µg/ml. This makes it significantly more potent than the standard antifungal drug Fluconazole, which had an MIC of 25 µg/ml and an MMC of 30 µg/ml. To our knowledge, this is the first report detailing the strong in vitro antimicrobial activity of a VA/ZnO/CS. Therefore, the synthesized VA/ZnO/CS is strongly supported as a highly effective, low-dose alternative for broad-spectrum antimicrobial applications. This suggests it may be a potential candidate for futuristic biomedical applications. Future work should further investigate the toxicity of VA/ZnO/CS using in vitro and in vivo studies on an animal model.

## Data Availability

The datasets generated during and/or analyzed during the current study are available from the corresponding author on reasonable request.

## Abbreviations

AMR: Antimicrobial resistance
ZnO NPs: Zinc oxide nanoparticles
VA/ZnO/CS: Vancomycin/zinc oxide/chitosan nanocomposite
UV–Vis: Ultraviolet–visible
FT-IR: Fourier transform-infrared spectroscopy
XRD: The x-ray diffraction
TEM: Transmission electron microscopy
MIC: Minimum inhibition concentration
MFC: Minimum fungicidal concentration
CFU: Colony-forming unit
ANOVA: One-way analysis of variance
SD: Standard deviation
